# The Effect of Ondansetron on Improvement of Symptoms in Patients with Irritable Bowel Syndrome with Diarrhea Domination: A Randomized Controlled Trial

**DOI:** 10.34172/mejdd.2024.386

**Published:** 2024-07-31

**Authors:** Sattar Jafari, Arezoo Atmani, Sepehr Gohari, Ehsan Seifi

**Affiliations:** ^1^Department of Internal Medicine, Zanjan University of Medical Sciences, Zanjan, Iran; ^2^Department of Internal Medicine, Vali-e-Asr Hospital, Zanjan University of Medical Sciences, Zanjan, Iran; ^3^Student Research Center, School of Medicine, Zanjan University of Medical Sciences, Zanjan, Iran; ^4^Department of Family Medicine, Alborz University of Medical Sciences, Alborz, Iran

**Keywords:** Irritable bowel syndrome, Diarrhea, Quality of life, Ondansetron, Randomized controlled trial

## Abstract

**Background:**

Diarrhea-dominant irritable bowel syndrome (IBS-D) is a deliberating and chronic condition that can impair social activities. Determining proper medication with satisfactory outcomes has been a challenge. The 5-hydroxytryptamine 3 receptor antagonist (5-HT3 RA) drugs have demonstrated favorable outcomes on IBS-D in the last 3 decades. Ondansetron, also a 5-HT_3_ RA is known as an antiemetic. Our aim was to evaluate the efficacy of ondansetron in IBS-D.

**Methods:**

In this single-center, double-blind, randomized controlled trial, patients with IBS-D were recruited. Patients were randomized on a 1:1 ratio and assigned into two groups: imipramine 25 mg/daily plus ondansetron 4 mg/3 times per day and imipramine 25 mg/daily plus placebo. The primary endpoint was the frequency of diarrhea per day after 8 weeks of treatment. The secondary endpoints consisted of changes in the frequency of defecation urgency per day, the number of days with gastrointestinal pain and bloating, and the patients’ overall satisfaction regarding bowel habits after 8 weeks of the treatment.

**Results:**

Data from 98 patients were analyzed. Ondansetron, compared to placebo, improved the primary outcome, and the stool consistency was increased significantly (3.29±1.19 vs 4.55±1.17, *P*<0.001). Moreover, the response rate for the diarrhea frequency was significantly higher in the ondansetron group compared to the placebo (77.5% vs 34.7%, *P*<0.001). In the ondansetron group, fewer urgencies were experienced, and pain severity and feeling of bloating declined as well (*P*<0.01).

**Conclusion:**

Ondansetron can mitigate almost all IBS-D-related symptoms, which may indicate it as a drug of choice; however, further evidence is required to ascertain its safety.

## Introduction

 Irritable bowel syndrome (IBS) is a functional bowel disorder characterized by changes in bowel movements and pain or abdominal discomfort in the absence of structural disorders.^1^ The pathophysiology of IBS is not well understood, but evidence of abnormal gastrointestinal (GI) motor function, visceral hypersensitivity, autonomic dysfunction, and psychological factors indicate disturbances within the enteric nervous system and the brain-gut axis.^2^ There is no definitive diagnostic indicator for IBS, and therefore, the diagnosis is based on clinical manifestations.^3^ The onset of the disease can occur at any age. The first symptoms, however, appear before age 45 in most cases.^4^ According to the main bowel dysfunction, it is categorized into three subtypes: (1) IBS-D (diarrhea dominant), (2) IBS-C (constipation dominant), and (3) IBS-M (mixed).^5^

 IBS-D is particularly a debilitating form of IBS as it reduces the quality of life due to the fear of pain, urgent defecation, and even incontinence.^6^ Serotonin or 5-hydroxytryptamine 3 (5-HT_3_) is an important neurotransmitter in the brain-gut axis and is involved in several functions of the GI tract, including the peristaltic reflex. At least seven different 5-HT receptor types have been described. 5-HT_3_ receptors are present both centrally and peripherally in the brain-gut axis, and 5-HT_3_ antagonists have been shown to reduce responses to noxious gut stimuli in animal studies.^7^ Some 5HT_3_-receptor antagonists (5HT3RA), such as ondansetron, cilansetron, and ramosetron, are useful in the treatment of IBS-D. Since 5-HT_3_ antagonists delay GI transit, the main adverse effect of this drug class is constipation.^8^ While earlier studies included IBS patients with non-constipated bowel habits (NC-IBS), later trials focused on diarrhea-predominant IBS (IBS-D).^9^ Ondansetron is a potent, highly selective 5HT3RA, which blocks 5HT_3_ receptors in the GI tract and in the central nervous system. Ondansetron is currently approved for use in adults and children for the management of nausea and vomiting induced by cytotoxic chemotherapy and radiotherapy, as well as for the prevention and treatment of postoperative nausea and vomiting. Constipation is an unintended side effect of ondansetron due to the decrement in colonic transit.^10,11^ In this randomized clinical trial (RCT) we aimed to investigate the effect of ondansetron on the symptoms of IBS-D.

## Methods

###  Study Design 

 This was a single-center, phase III, double-blind, randomized, placebo-controlled trial to assess the effect of ondansetron (4 mg tablet/three times daily) versus placebo for 8 weeks in patients with IBS-D.

###  Study Objective

 The objective of this RCT was to test the hypothesis of the beneficial effects of ondansetron on the improvement of IBS-D symptoms after 8 weeks of the treatment.

###  Trial Population and Eligibility Assessment

 Our goal was to randomize 100 eligible patients from the gastroenterology clinic of Vali-e-asr Hospital, Zanjan, Iran, from June 2019 to March 2020. Patients with IBS-D and aged between 18-50 years old were eligible to be recruited. The diagnosis of IBD was made using ROME IV criteria: The recurrent abdominal pain ≥ 1 day/wk in the last 3 months (on average) associated with two or more of the following: (1) Defecation (Either increasing or improving), (2) A change in stool frequency and (3) A change in stool form (appearance). The exclusion criteria were: (1) pregnancy and lactation, (2) prior intestinal surgery except for appendectomy and cholecystectomy, (3) prior known inflammatory bowel disease (IBD), (4) anti-psychotic or intestinal associated drugs (except imipramine which was assigned for both groups), (5) drugs that interact with ondansetron metabolism including P450 enzyme inducer/inhibitor, and (6) patients’ decline to participate in the study.

###  Randomization, Intervention and Follow-up

 The randomization method employed in this study was permuted block randomization based on quaternary blocks. During the recruitment phase, eligible patients were randomized by sex and age and then assigned into two intervention groups (1 and 2). The first arm received imipramine tablets 25 mg/daily (Abidi Pharma Company^®^) plus ondansetron tablets 4 mg/3 times per day, and the other arm received imipramine tablets 25mg/daily (Abidi Pharma Company^®^) plus placebo tablets containing Avicel 4 mg/3 times per day. The treatment duration was 8 weeks. Before the initiation of the designated drug regimen, imipramine tablet 10 mg/daily (Abidi Pharma Company^®^) was prescribed for both study arms, and after 1 week, the drug dosage increased to 25 mg/daily. All patients were followed up bimonthly by office visits, during which the patients’ adherence to the treatment was evaluated, and physical examination and complete medical history were conducted to assess the possible adverse events. Patients were recommended to attend the instructed visits even after premature discontinuation from the trial. Moreover, patients were instructed to return all used and unused medication at the end of week 8.

###  Blinding

 Ondansetron tablets with the same shape, color, and package with different code combinations were used for two study arms. After recruitment, a specific code was assigned to each patient, which remained the same until the end of the study. Patients, investigators, and outcome assessors were blinded to the assigned treatment to each intervention group. The un-blinded treatment list was held by the Zanjan University Medical Council, and in case of an urgency for un-blinding, the Research Council of Zanjan University was notified, and ultimately, the patient was sent to an external physician for further assessments.

###  Outcomes

####  Primary Outcome

 The primary outcome was the changes in the frequency of diarrhea per day after 8 weeks of treatment between the two study arms. The translated Bristol stool scale form and IBS severity score questionnaire were used to measure the primary outcome.^12,13^

####  Secondary Outcomes

 The secondary outcomes included (1) changes in the frequency of defecation urgency per day after 8 weeks of treatment between the two study arms, (2) changes in the frequency of the days with GI pain during 8 weeks of treatment between the two study arms, (3) changes in the frequency of the days with GI bloating during 8 weeks of treatment between two study arms, and (4) patient’s overall satisfaction regarding the bowel habits after 8 weeks of the treatment between two study arms. All of the secondary outcomes were measured using IBS severity score questionnaire.

###  Study Monitoring and Adverse Event Recording

 The Research Council and Ethics Committee of Zanjan University of Medical Sciences were responsible for monitoring and data verification of the study. All the forms used in the study, whether translated or in original language, were approved by the mentioned committee before receiving the ethics code. The study was monitored by the research council monitoring team via random visits during each phase of the study. Any adverse event, related or unrelated, was recorded in the standard adverse event form.

###  Statistical Consideration

####  Sample Size Calculation

 Based on the results of the study conducted by Garsed and colleagues^14^ regarding the effect of ondansetron on the symptoms of IBS, the sample size was calculated as below:


$$n=\frac{\left\{1-\frac{\alpha }{2}\sqrt{\bar{P}\left(1-\bar{P}\right)}+Z_{1-\beta }\sqrt{P_{1}\left(1-P_{1}\right)+P_{2}\left(1-P_{2}\right)}\right\}}{{\left(P_{1}-P_{2}\right)}^{2}}$$

Where n is number of patients in each group, P_1_ = 0.17 and P_2_ = 0.43. With a study power of 80%, an alpha level of 0.05%, and 10% loss, the sample size was calculated equal to 55 patients in each group. As our study aimed to investigate the effect of ondansetron only on IBS-D, patients with IBS-C were excluded during the sample size calculation.

####  Statistical Analysis Plan

 To interpret the results, mean and standard deviation were used for quantitative data, and frequency and percent were used for qualitative data. The per-protocol approach was implemented to analyze the data. For the assessment of the normality distribution of continuous quantitative data, the Kolmogorov–Smirnov test was used. The independent *t*-test was used for the analysis of quantitatively continuous variables on condition of normal distribution. Otherwise, the Mann-Whitney U test was used. Multivariate analysis was used to mediate the effect of confounding factors (if any presented). The primary outcome was considered confirmatory, and the secondary outcomes were considered as exploratory as the sample size was not calculated for them. A *P* value < 0.05 was considered statistically significant. All the data were analyzed using SPSS software (version 24.0, SPSS, Chicago, Illinois).

## Results

###  Demographic Data and Baseline Characteristics

**Figure 1:**
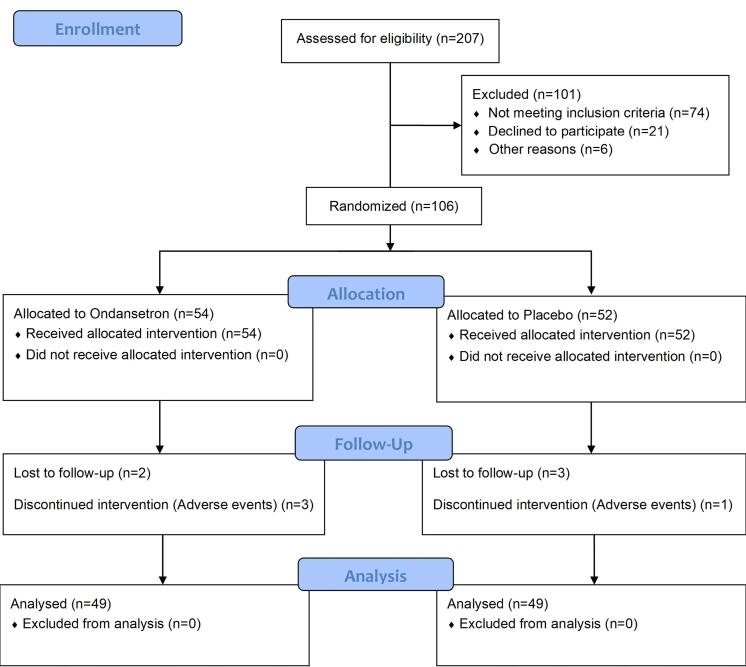
Consort flow diagram

 After the eligibility assessment, 207 patients were eligible to participate in the study. During the eligibility assessment, 101 patients were excluded (74 did not meet inclusion criteria, 21 declined to participate, and six were excluded due to other reasons such as the investigators’ decision on patients’ poor capacity to stick to the intervention), and 106 (54 in the drug and 52 in the placebo group) patients were recruited. After the study period, the data from 98 patients were analyzed (Figure 1). The mean ± SD of age was 32.16 ± 8.82 and 31.02 ± 8.05 in ondansetron and placebo arms, respectively. In both arms, 17 (34.7%) patients were men. Patients were well-balanced according to age and sex (*P* > 0.05). Also, according to the checklist results, there was no significant difference in residency location and level of education between the two groups (*P* > 0.05, Table 1). According to the IBS severity score questionnaire results, there was no significant difference regarding the obtained scores between the two groups (*P* > 0.05) except for the effect of IBS on quality of life (QoL), which had a higher score in the drug group (*P* = 0.017, Table 2).

**Table 1:** Demographic and baseline characteristics

**Variable**		**Ondansetron** **(n=49)**	**Placebo** **(n=49)**	***P*** ** value**
Gender	Male, n (%)	17 (34.7)	17 (34.7)	NS
	Female, n (%)	32 (65.3)	32 (65.3)	
Age		32.16 ± 8.82	31.02 ± 8.05	NS
Residency status	Urban, n (%)	42 (85.7)	43 (87.8)	NS
	Rural, n (%)	7 (14.3)	6(12.2)	
Literacy status	Illiterate, n (%)	1 (2.0)	0 (0.0)	NS
	Under educated, n (%)	6 (12.2)	7 (14.3)	
	High school diploma, n (%)	11 (22.4)	14 (28.6)	
	University degree, n (%)	31 (63.3)	28 (57.1)	

**Table 2:** The results of the irritable bowel syndrome severity score questionnaire at baseline

**Variable**		Ondansetron* **(n=49)**	Placebo* **(n=49)**	***P*** ** value**
Diarrhea frequency per day, n (%)	< 3	3 (6.1)	5 (10.2)	0.435
	3-6	21 (42.9)	25 (51.0)	
	6 <	25 (51.0)	19 (38.8)	
Defecation urgency per day, n (%)	< 3	7 (14.3)	12 (24.5)	0.183
	3-5	21 (42.9)	24 (49)	
	5 <	21 (42.9)	13 (26.5)	
Days with gastrointestinal pain, n (%)	< 3	1 (2.0)	2 (4.1)	0.427
	3-5	13 (26.5)	18 (36.7)	
	5-7	35 (71.4)	29 (59.2)	
Days with bloating, n (%)	< 3	4 (8.2)	5 (10.2)	0.899
	3-5	13 (26.5)	14 (28.6)	
	5-7	32 (65.3)	30 (61.2)	
Pain intensity, n (%)	0	0 (0.0)	0 (0.0)	0.153
	1	3 (6.1)	4 (8.2)	
	2	15 (30.6)	19 (38.8)	
	3	13 (26.5)	18 (36.7)	
	4	18 (36.7)	8 (16.3)	
Bloating intensity, n (%)	0	1 (2.0)	0 (0.0)	0.258
	1	1 (2.0)	6 (12.2)	
	2	14 (28.6)	12 (24.5)	
	3	12 (24.5)	14 (28.6)	
	4	21 (42.9)	17 (34.7)	
Bowel habit satisfaction, n (%)	1	22 (44.9)	20 (40.8)	0.683
	2	27 (55.1)	29 (59.2)	
	3	0 (0.0)	0 (0.0)	
	4	0 (0.0)	0 (0.0)	
Effect of IBS on quality of life, n (%)	0	0 (0.0)	0 (0.0)	0.017
	1	0 (0.0)	7 (14.3)	
	2	24 (49.0)	24 (49.0)	
	3	25 (51.0)	18 (36.7)	

IBS: Irritable bowel syndrome. * Based on the per-protocol approach implementation, only patients who had finished the study included in the analysis and the report.

###  Outcomes

####  Primary Outcome Analysis

 After the treatment period, the frequency of diarrhea per day was reduced in both groups. The drug response rate for the frequency of diarrhea in the intervention group was 77.5% (38/49), and for the placebo was 34.7% (17/49). Ondansetron plus imipramine was superior compared with imipramine alone in the reduction of the frequency of diarrhea per day (*P <*0.001, Table 3). Also, the Bristol stool scale was significantly lower in the drug group compared with the placebo (3.29 ± 1.19 vs 4.55 ± 1.17, *P* < 0.001, Table 4), and patients’ stool in the drug group had higher consistency. Also, the response rates for the stool consistency in the ondansetron and placebo groups were 48.9% (24/49) and 26.53% (13/49) (*P* < 0.01), respectively.

**Table 3:** The results of the irritable bowel syndrome severity score questionnaire at week 8

**Variable**		**Ondansetron** **(n=49)**	**Placebo** **(n=49)**	***P*** ** value**
Diarrhea frequency per day, n (%)	< 3	41 (83.7)	22 (44.9)	0.001
	3-6	8 (16.7)	27 (55.1)	
	6 <	0 (0.0)	0 (0.0)	
Defecation urgency per day, n (%)	< 3	48 (98.0)	25 (51.0)	0.001
	3-5	1 (2.0)	24 (49.0)	
	5 <	0 (0.0)	0 (0.0)	
Days with gastrointestinal pain, n (%)	< 3	36 (73.5)	21 (42.9)	0.002
	3-5	13 (26.5)	28 (57.1)	
	5-7	0 (0.0)	0 (0.0)	
Days with bloating, n (%)	< 3	26 (53.1)	9 (18.4)	0.001
	3-5	22 (44.9)	33 (67.3)	
	5-7	1 (2.0)	7 (14.3)	
Pain intensity, n (%)	0	18 (36.7)	4 (8.2)	< 0.001
	1	29 (59.2)	33 (67.3)	
	2	2 (4.1)	12 (24.5)	
	3	0 (0.0)	0 (0.0)	
	4	0 (0.0)	0 (0.0)	
Bloating intensity, n (%)	0	8 (16.3)	3 (6.1)	0.014
	1	27 (55.1)	17 (34.7)	
	2	11 (22.4)	26 (53.1)	
	3	3 (6.1)	3 (6.1)	
	4	0 (0.0)	0 (0.0)	
Bowel habit satisfaction, n (%)	1	0 (0.0)	0 (0.0)	< 0.001
	2	3 (6.1)	20 (40.8)	
	3	28 (57.1)	4 (49.0)	
	4	18 (36.7)	5 (10.2)	
Effect of IBS on quality of life, n (%)	0	9 (18.4)	3 (6.1)	0.291
	1	20 (40.8)	21 (42.9)	
	2	19 (38.8)	23 (46.9)	
	3	1 (2.0)	2 (4.1)	

IBS: Irritable bowel syndrome

**Table 4:** Changes of Bristol stool scale between the study arms at baseline and week 8

**Variable**	**Ondansetron (n=49)**	**Placebo (n=49)**	***P*** ** value**
	**Mean±SD**	**Mean±SD**	
Baseline	6.57 ± 0.50	6.59 ± 0.54	0.846
Week 8	3.29 ± 1.09	4.55 ± 1.17	< 0.001

####  Secondary Outcomes Analysis

 Based on the results of the IBS severity score questionnaire, all of the scores in each item of the questionnaire were improved from baseline in each study arm. However, the drug group was superior in all questionnaire items compared with the placebo group (*P* < 0.05), except for the quality of life statement (*P* = 0.291). In detail, after the treatment period, almost all the patients in the drug group were somehow satisfied with the bowel habits (cumulative rate of satisfaction: 93.8%); on the other hand, slightly more than half of the patients were satisfied with bowel habits in the placebo group (cumulative rate of satisfaction: 59.2%). Like the bowel satisfaction level, after the treatment period, almost all the patients (98%) in the drug group had less than three episodes of emergency defecation per day, which was 51% in the placebo group (*P* < 0.001) (Table 3).

###  Adverse Events

 During the study period, no severe adverse event was reported. Five patients in the drug group developed constipation and dry mouth, and three of them refused to continue the trial. One patient in the placebo group developed a headache and discontinued the drug.

## Discussion

 Ondansetron was primarily introduced for the therapeutic purpose of chemotherapy-induced nausea and vomiting; however, its influence on colonic transit disclosed it as an anti-diarrhea agent. Moreover, the effect of ondansetron in IBS-D has been assessed by further trials.^14,15^ Patients with IBS have altered gut function and 5-HT signaling that can result in abdominal pain and urgency.^16^ 5-HT3 receptor antagonists such as ondansetron are a group of drugs that confer an inhibitory action on 5-HT3 receptors in mucosal processes of enteric afferent neurons and attenuate motor activity; thus, they can amend visceral hypersensitivity.^17,18^ The extensive availability of ondansetron compared with other drugs in its class, such as alosetron, ramosetron, and cilansetron, has led to its broader use through the years.^19^ Alosetron, also a 5-HT3 RA, was shown to have substantial benefits. Unfortunately, it was withdrawn due the reported evidence regarding the unacceptable increased risk of severe constipation and a much lower incidence of ischemic colitis.^20^ Ramosetron is another potent drug member of 5-HT3 RA for non-constipated IBS that has recently proved to be effective regardless of sex.^9^

 This double-blind, randomized controlled trial presents compelling results on the efficacy of 4 mg/three times daily of ondansetron as an effective medication in patients with IBS-D. By the end of 8 weeks, the drug group reported significantly more favorable Bristol stool scales in addition to a significantly decreased frequency rate of diarrhea. More frequent bowel movements and shorter colonic transit time are the abnormal components of GI motility in IBS-D patients.^21^ Although the principal difference from normal is the day-to-day variability, one of the most commonly stated triggers for this variability is stress.^22^ IBS-D patients are commonly affected by psychological stresses, which can stimulate colon transit and motility, possibly by means of 5-HT3 release.^23^ There are some cited abnormalities in mucosal 5-HT3 metabolism in IBS-D; several studies of humans and animals demonstrated that mucosal biopsies from IBS patients released more spontaneous 5-HT3.^24,25^ Therefore, a blockage by 5-HT3 RA, like ondansetron, is likely related to the pathophysiology of IBS. Ondansetron belongs to the category of selective serotonin receptor antagonists, which is designed to target serotonin pathways. By depleting 5-HT3 from neurons, ondansetron contributes to abolishing colonic migrating motor complexes and as a consequence, lowers the intestinal transit.^17^ Hence, considerable improvements in stool consistency in our patients have resulted. Previous trials evaluated the effects of immediate release and bimodal ondansetron have inferred similar results.^14,25^ In a phase II trial, Plasse and colleagues showed that ondansetron, compared with placebo, has a significant superiority in stool consistency, with a response rate of 56% vs 35.3%. Although an additional superiority was seen on reduction of pain in the ondansetron group, the difference was not significant.^26^ Unlike the former studies, intensity and duration of GI pain in our study decreased significantly with ondansetron. Seemingly, 5-HT3 RAs are able to alleviate pain via inhibiting the spinal pathways that mediate the response to painful colonic distention.^27^

 In contrast with the mentioned studies, a higher response rate was observed in this study regarding the frequency of diarrhea in both ondansetron and placebo groups. The probable explanation may be attributed to the administration of imipramine for both groups from baseline. It has been shown that tricyclic antidepressant drugs reduce GI transit.^28^ Consequently, starting imipramine in this trial could explain the higher response rate in both groups. The stool consistency, however, may not be influenced by imipramine as the result of this study was consistent with other trials.

 Since constipation is an expected side effect of ondansetron that may interfere with the quality of life,^29^ we chose a relatively cautious daily dose of ondansetron to prevent the undesirable side effects; therefore, patients expressed more significant satisfaction in their bowel habits.

 We did not detect a significant difference regarding the QoL between the study arms at the end of treatment, which is in line with the study of Garsed et al.^1^ Although, advances in IBS-QoL were more prominently shown in alosetron and ramosetron trials.^9,30^

## Limitations of the Study

 Our study has some limitations: 1) we did not observe notable side effects of ondansetron. More studies are needed to investigate the incidence rate and severity of side effects. 2) Since there are no official dose recommendations for ondansetron in IBS, future studies may consider administering several different doses to attain the optimal effect level with the least probable adverse outcomes. 3) As the symptoms of IBS can fluctuate during the period of time and may symptom relief occur during a certain time without any intervention, thus further trials with an extended duration of follow-up are needed to cover this issue. 4) We used a per-protocol approach for the outcome analysis, and data of participants in the same group that they were originally allocated were not included, which may have influenced our results. 5) The concomitant use of imipramine with the ondansetron may also interact with the results.

## Conclusion

 Given the reduction in diarrhea episodes and urgency, less abdominal pain, improved bloating, and stool consistency, important implications are provided for clinicians to address IBS-D symptoms more promptly. Ondansetron seems to be a promising treatment alternative for patients with IBS-D as it has an acceptable safety profile and widespread availability at a reasonable cost.

## References

[1] Chey WD, Kurlander J, Eswaran S (2015). Irritable bowel syndrome: a clinical review. JAMA.

[2] Holtmann GJ, Ford AC, Talley NJ (2016). Pathophysiology of irritable bowel syndrome. Lancet Gastroenterol Hepatol.

[3] Moayyedi P, Mearin F, Azpiroz F, Andresen V, Barbara G, Corsetti M (2017). Irritable bowel syndrome diagnosis and management: a simplified algorithm for clinical practice. United European Gastroenterol J.

[4] Pimentel M, Lembo A (2020). Microbiome and its role in irritable bowel syndrome. Dig Dis Sci.

[5] Su AM, Shih W, Presson AP, Chang L (2014). Characterization of symptoms in irritable bowel syndrome with mixed bowel habit pattern. NeurogastroenterolMotil.

[6] Lembo AJ, Lacy BE, Zuckerman MJ, Schey R, Dove LS, Andrae DA (2016). Eluxadoline for irritable bowel syndrome with diarrhea. N Engl J Med.

[7] Gale JD, Bunce KT. Pharmacological characterization of 5-hydroxytryptamine receptors in the gastrointestinal tract. In: Serotonin and Gastrointestinal Function. CRC Press; 2020. p. 33-52.

[8] Park YS, Sung KW (2019). Gastroprokinetic agent, mosapride inhibits 5-HT3 receptor currents in NCB-20 cells. Korean J PhysiolPharmacol.

[9] Fukudo S, Ida M, Akiho H, Nakashima Y, Matsueda K. Effect of ramosetron on stool consistency in male patients with irritable bowel syndrome with diarrhea. Clin Gastroenterol Hepatol 2014;12(6):953-9.e4. 10.1016/j.cgh.2013.11.024. 24315882

[10] Vetrivel N, Praveen D, Ranadheer Chowdary P, Elan Cheziyan K, Vijey Aanandhi M (2018). Comparison of safety and effectiveness of ondansetron and domperidone in patients with gastrointestinal problems. Drug Invention Today.

[11] Ryu J, So YM, Hwang J, Do SH (2010). Ramosetron versus ondansetron for the prevention of postoperative nausea and vomiting after laparoscopic cholecystectomy. Surg Endosc.

[12] Blake MR, Raker JM, Whelan K (2016). Validity and reliability of the Bristol Stool Form Scale in healthy adults and patients with diarrhoea-predominant irritable bowel syndrome. Aliment PharmacolTher.

[13] Francis CY, Morris J, Whorwell PJ (1997). The irritable bowel severity scoring system: a simple method of monitoring irritable bowel syndrome and its progress. Aliment PharmacolTher.

[14] Garsed K, Chernova J, Hastings M, Lam C, Marciani L, Singh G (2014). A randomised trial of ondansetron for the treatment of irritable bowel syndrome with diarrhoea. Gut.

[15] Maxton DG, Morris J, Whorwell PJ (1996). Selective 5-hydroxytryptamine antagonism: a role in irritable bowel syndrome and functional dyspepsia?. Aliment PharmacolTher.

[16] Crowell MD, Shetzline MA, Moses PL, Mawe GM, Talley NJ (2004). Enterochromaffin cells and 5-HT signaling in the pathophysiology of disorders of gastrointestinal function. CurrOpinInvestig Drugs.

[17] Spencer NJ, Nicholas SJ, Sia TC, Staikopoulos V, Kyloh M, Beckett EA (2013). By what mechanism does ondansetron inhibit colonic migrating motor complexes: does it require endogenous serotonin in the gut wall?. NeurogastroenterolMotil.

[18] Goldberg PA, Kamm MA, Setti-Carraro P, van der Sijp JR, Roth C (1996). Modification of visceral sensitivity and pain in irritable bowel syndrome by 5-HT3 antagonism (ondansetron). Digestion.

[19] Black CJ, Ford AC, Houghton LA (2019). Editorial: understanding differences in patient response to ondansetron in irritable bowel syndrome with diarrhoea-are we any closer?. Aliment PharmacolTher.

[20] Chang L, Chey WD, Harris L, Olden K, Surawicz C, Schoenfeld P (2006). Incidence of ischemic colitis and serious complications of constipation among patients using alosetron: systematic review of clinical trials and post-marketing surveillance data. Am J Gastroenterol.

[21] Chey WY, Jin HO, Lee MH, Sun SW, Lee KY (2001). Colonic motility abnormality in patients with irritable bowel syndrome exhibiting abdominal pain and diarrhea. Am J Gastroenterol.

[22] Hertig VL, Cain KC, Jarrett ME, Burr RL, Heitkemper MM (2007). Daily stress and gastrointestinal symptoms in women with irritable bowel syndrome. Nurs Res.

[23] Spiller RC (2011). Targeting the 5-HT3 receptor in the treatment of irritable bowel syndrome. CurrOpinPharmacol.

[24] Keating C, Beyak M, Foley S, Singh G, Marsden C, Spiller R (2008). Afferent hypersensitivity in a mouse model of post-inflammatory gut dysfunction: role of altered serotonin metabolism. J Physiol.

[25] Cremon C, Carini G, Wang B, Vasina V, Cogliandro RF, De Giorgio R (2011). Intestinal serotonin release, sensory neuron activation, and abdominal pain in irritable bowel syndrome. Am J Gastroenterol.

[26] Plasse TF, Barton G, Davidson E, Abramson D, Kalfus I, Fathi R (2020). Bimodal release ondansetron improves stool consistency and symptomatology in diarrhea-predominant irritable bowel syndrome: a randomized, double-blind, trial. Am J Gastroenterol.

[27] Färber L, Haus U, Späth M, Drechsler S (2004). Physiology and pathophysiology of the 5-HT3 receptor. Scand J Rheumatol Suppl.

[28] Grover M, Camilleri M (2013). Effects on gastrointestinal functions and symptoms of serotonergic psychoactive agents used in functional gastrointestinal diseases. J Gastroenterol.

[29] Gunn D, Fried R, Lalani R, Farrin A, Holloway I, Morris T (2019). Treatment of irritable bowel syndrome with diarrhoea using titrated ondansetron (TRITON): study protocol for a randomised controlled trial. Trials.

[30] Cremonini F, Nicandro JP, Atkinson V, Shringarpure R, Chuang E, Lembo A (2012). Randomised clinical trial: alosetron improves quality of life and reduces restriction of daily activities in women with severe diarrhoea-predominant IBS. Aliment PharmacolTher.

